# Asymmetric Dimethylarginine: A Never-Aging Story

**DOI:** 10.1055/a-2537-4692

**Published:** 2025-05-26

**Authors:** Natalia Jarzebska, Stefan R. Bornstein, Sergey Tselmin, Ulrich Julius, Barbara Cellini, Richard Siow, Mike Martin, Rajeshwar P. Mookerjee, Arduino A. Mangoni, Norbert Weiss, Roman N. Rodionov

**Affiliations:** 1 Department of Internal Medicine III, University Hospital Carl Gustav Carus at the Technische Universität Dresden, Dresden, Germany; 2 School of Cardiovascular and Metabolic Medicine and Sciences, Faculty of Life Sciences & Medicine, King’s College London, London, United Kingdom of Great Britain and Northern Ireland; 3 Department of Medicine and Surgery, University of Perugia, Perugia, Italy; 4 Ageing Research at King’s (ARK), King’s College London, London, United Kingdom of Great Britain and Northern Ireland; 5 Department of Physiology, Anatomy and Genetics, Medical Sciences Division, University of Oxford, Oxford, United Kingdom of Great Britain and Northern Ireland; 6 Department of Psychology, University of Zurich, Zurich, Switzerland; 7 Healthy Longevity Center, University of Zurich, Zurich, Switzerland; 8 Institute of Liver and Digestive Health, University College London, London, United Kingdom of Great Britain and Northern Ireland; 9 Department of Clinical Pharmacology, College of Medicine and Public Health, Flinders University and Flinders Medical Centre, Adelaide, Australia

**Keywords:** asymmetric dimethylarginine, anti-aging therapy, therapeutic apheresis

## Abstract

Human aging is intrinsically associated with the onset and the progression of several disease states causing significant disability and poor quality of life. Although such association was traditionally considered immutable, recent advances have led to a better understanding of several critical biochemical pathways involved in the aging process. This, in turn, has stimulated a significant body of research to investigate whether reprogramming these pathways could delay the progression of human ageing and/or prevent relevant disease states, ultimately favoring healthier aging process. Cellular senescence is regarded as the principal causative factor implicated in biological and pathophysiological processes involved in aging. Asymmetric dimethylarginine (ADMA) is an endogenous inhibitor of nitric oxide synthase and an independent risk factor for several age-associated diseases. The selective extracorporeal removal of ADMA is emerging as a promising strategy to reduce the burden of age-associated disease states. This article discusses the current knowledge regarding the critical pathways involved in human aging and associated diseases and the possible role of ADMA as a target for therapies leading to healthier aging processes.

## Introduction

The rapid change in age distribution represents the most important phenomenon in our society globally. In 2019, about 9% of people worldwide were 65 years and older, and it is estimated that in 2050 one in every six people will be over the age of 65 1 . This demographic transition requires changes in resource allocation and delivery of health care as increased longevity is not necessarily associated with an extended time in good health 2 . Therefore, disease prevention and maintenance of an acceptable level of quality of life and independence in advanced age has become a major public health issue and research focus of gerontology 3 . The process of aging is generally characterized by a gradual functional decline and reduced homeostatic capacity. In mammals, this process is highly heterogeneous, which further adds to the challenges of managing this complex patient group, typically characterized by the coexistence of several disease states.

Multiple epidemiological studies identified the endogenous analogue of L -arginine asymmetric dimethylarginine (ADMA) as an independent predictor of morbidity and mortality in the elderly 4 . Furthermore, elevated levels of ADMA were shown to be associated with adverse outcomes in patients with age-related diseases, such as cardiovascular disease 5 , chronic kidney disease 6 , and peripheral artery disease 7 . What is more, experiments in animal models have convincingly shown the protective effects of ADMA lowering strategies in multiple models of acute and chronic cardiovascular and metabolic injury. The goal of our manuscript is to summarize the role of ADMA as a critical marker and mediator of age-associated pathologies and to discuss promising ADMA lowering strategies favoring healthier aging and increased life expectancy.

## Age-related diseases

For several decades it has been suggested that there may be an undervalued, but nevertheless very important link between human aging and many chronic disorders and that aging increases the risk of many common diseases, including cardiovascular disease 8 , dementia 9 , osteoporosis 10 , osteoarthritis 11 , type 2 diabetes 12 , idiopathic pulmonary fibrosis 13 , glaucoma 14 , Alzheimer’s disease 15 , and metabolic-associated fatty liver disease (MAFLD) 16 . Moreover, older patients often suffer from multiple comorbidities requiring combinations of different treatments. However, this increases the risk of drug-drug and drug-disease interactions with consequent reduced treatment efficacy and increased toxicity 17 . In recent years, inflammaging has emerged as a key concept in the field of gerontology. It is now widely recognized that aging is associated with a state of increased pro-inflammatory markers and dysregulated immune responses, which contribute to the development and progression of age-related diseases 18 .

Since human aging is closely interconnected with the pathophysiology of several chronic diseases, understanding the molecular mechanisms underpinning the aging process is likely to facilitate the identification of novel druggable targets for diseases associated with advanced age.

## Cellular senescence and other mechanisms of aging

**Fig. 1:**
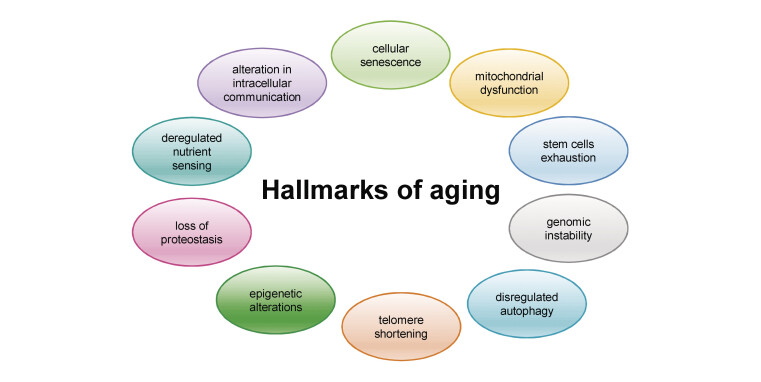
Hallmarks of aging. Ten common cellular and molecular traits generally considered to contribute to the process of aging and together determining the aging phenotype.

Our understanding of aging remains limited, and its biological causes are largely unknown. However, recent studies have led to the identification of common molecular traits associated with aging, collectively called aging hallmarks, including telomere shortening, mitochondrial dysfunction, cellular senescence, deregulated nutrient sensing, loss of proteostasis, epigenetic alterations and genomic instability, stem cell exhaustion, and alterations in intracellular communication 19 . Over the years, autophagy was also added as the 10th hallmark ( Fig. 1 ) 20 21 . Their recognition has stimulated a considerable body of research to prevent or delay the onset of age-related diseases by reprogramming the aging process itself. The rapidly increasing knowledge regarding the molecular mechanisms of aging has also changed people’s attitudes towards this process. Traditionally, aging was seen as an unavoidable and unchangeable process determined by genetic programs or accidental events ultimately leading to death. However, it was later discovered that the speed of aging can be to some extent modified, for example, by light intensity in Drosophila 22 , or by caloric restriction in mice and rats 23 24 . The results of these studies led to a reconsideration of aging as a modifiable process that can possibly be influenced even at the molecular level 25 . The current state of knowledge on the mechanisms of aging is elegantly described in a review by Guo and colleagues 26 . Here, we focus mainly on cellular senescence, a principal causative factor facilitating aging and aging-associated diseases 26 27 .

Cellular senescence, first described by Hayflick and Moorhead in 1961 in cultured human fibroblasts 28 , is defined as a terminal and permanent state of growth arrest, in which cells are unable to proliferate in spite of mitogenic stimuli and optimal growth conditions. Therefore, cellular senescence represents the critical mechanism underpinning tissue ageing, a condition where cells progressively lose their ability to proliferate, consequently replacing damaged cells that otherwise accumulate 29 . Although the contribution of cellular senescence to aging has been long suspected, it was confirmed only recently. Studies investigating the rapidly aging BubR1 (a protein that ensures proper segregation of chromosomes during mitosis) hypomorphic mouse model showed that decreased levels of this protein led to a variety of early aging characteristics, including reduced lifespan, cataracts, lipodystrophy and infertility very early in life 30 and p16 (cyclin-dependent kinase inhibitor) tissue accumulation 31 . In the absence of p16, the age-related phenotype was attenuated, clearly demonstrating the direct relation of cellular senescence and aging. It has later been shown also in other mice models that removing cells expressing p16 is beneficial in age-related diseases, including Parkinson’s disease 32 , Alzheimer’s disease 33 , atherosclerosis 34 , and osteoarthritis 35 . Senescent cells are resistant to apoptosis owing to upregulation of molecular pathways involved in cell survival, including the BCL-2 (regulator of apoptosis) family of proteins 36 . The precise mechanisms determining whether a cell undergoes senescence or apoptosis are not fully understood, but possibly depend on the nature, duration and intensity of the stimulus, as well the cell type 37 . A number of stimuli inducing senescence are discussed in the following sections.

## DNA damage response

Damage to nuclear DNA, mainly in the form of DNA double-strand breaks (DSBs) 38 is considered one of the most important factors facilitating cellular senescence. DSBs activate the DNA damage response (DDR) pathway, which is supposed to block the cell cycle to prevent the propagation of damaged genetic information. If the damage cannot be ameliorated by repair mechanisms prolonged DDR signaling results in senescence 39 . This concept is supported by the fact that inhibition of DDR signaling kinases (ATM – Ataxia-telangiectasia mutated, ATR – ataxia telangiectasia and Rad3-related, CHK1 – checkpoint kinase 1 and CHK2 – checkpoint kinase 2) restores the capacity of previously senescent cells to enter the cell cycle 40 41 . The tumor suppressor p53 is at the bottom of the DDR signaling cascade and its activation stimulates the expression of cyclin-dependent kinase inhibitor p21, an important mediator of cell cycle arrest associated with senescence 42 . Another inhibitor, p16 is activated later in the process, presumably to sustain the senescence phenotype 43 .

## Telomere shortening

One of the first recognized and best characterized mechanisms leading to cellular senescence is telomere shortening. When the standard DNA duplication machinery is unable to fully duplicate the ends of the chromosomes and the telomere maintenance mechanisms are absent, telomeres are shortened during each round of DNA replication. Below a certain length, the loss of telomere-capping factors, other protective structures, or even one or a few telomeres leads to the activation of DDR through a pathway involving ATM, p53, and p21, which in turn triggers replicative cellular senescence 44 .

## Dysregulated autophagy

Autophagy, along with apoptosis and necrosis, is a common type of cell death. It is a process that involves the degradation of organelles and proteins to remove cellular debris and sustain physiological cell functions 45 . Autophagy is tightly regulated and the proteins that need to be degraded through this pathway are ubiquitinated and engulfed by the phagophore, which later forms an autophagosome 46 . Several studies in animal models have demonstrated that the maintenance of proper autophagic activity is associated with extended longevity 47 48 49 . By contrast, disabled autophagy is considered as one of the primary hallmarks of aging 19 and it has been shown to decrease life and health span in lower organisms 50 51 . Recently, similar data were reported in mice models, providing evidence that autophagy modulates longevity also in mammals 52 53 54 . Both a reduction and an increased autophagy are strongly associated with aging and age-related diseases. Previous investigations have shown that autophagy decreases with age, potentially contributing to the accumulation of damaged organelles, metabolic alterations in the cells, and decreased lysosomal proteolytic activity 47 55 . On the other hand, uncontrolled or dysregulated autophagy might also accelerate aging by increasing the number of senescent cells, causing fast muscle fibers atrophy, cardiac hypertrophy, sarcopenia, neurodegeneration, and molecular and metabolic dysregulation 55 56 .

## Mitochondrial dysfunction and reactive oxygen species

Dysfunctional mitochondria may play an important role in senescence as this process is induced by the chemical inhibition of mitochondrial function 57 . Alterations of mitochondrial homeostasis have also been shown to drive age-dependent modifications 58 59 60 . Ineffective control of reactive oxygen species (ROS) on mitochondrial supercomplexes causes changes in ROS signaling, leading to cellular stress and age-dependent damage. High levels of mitochondrial ROS significantly contribute to aging, as shown in superoxide dismutase-deficient mice (SOD1-KO and SOD3-KO) 61 62 .

In summary, human lifespan is closely related to the reduction of the regenerative potential of tissues and organs. The aging process is driven by a number of complex molecular pathways, which taken together prevent cell proliferation, alter metabolism and gene expression patterns and induce high levels of reactive oxygen species, ultimately maintaining the cellular senescent phenotype. Even though the amount of early senescent cells is low, they can effectively limit the regenerative potential of tissue stem cells and induce the accumulation of cellular damage, leading to age-related diseases 63 .

## Healthy aging

Healthy aging is defined as “the process of developing and maintaining the functional ability that enables well-being in older age” 64 . Maintenance of good health in advanced age has become a major public health challenge. The impact of lifestyle on health status is well established. Dietary habits are one of the key modifiable lifestyle factors for the prevention and/or amelioration of age-associated diseases and the maintenance of healthy aging 65 . Even though the evidence on healthy aging differs by various dietary patterns, it seems that dietary habits focused on plant-based foods promote healthy aging by positively influencing cognition, psychological function, sensory function, and motility. Current recommendations based on a high consumption of fruits, vegetables, whole grains, moderate consumption of dairy, fish and poultry, and low consumption of red meat, saturated fat and sugars are in line with these observations 64 . Although caloric restriction (CR) is not a dietary pattern itself, avoiding excess intake of calories can be applied to any diet and is common in eating habits that are associated with reduced risk of age-linked diseases and improved longevity. In particular, CR refers to the reduction in the total energy intake by 20 to 40%, but without leading to malnutrition or deficiency in essential nutrients. In experimental studies, CR has been shown to increase life expectancy and delay the onset and progression of multiple age-associated diseases in diverse species. In humans, however, long-term CR has been associated with both beneficial and detrimental effects 66 67 68 . Caloric restriction has demonstrated beneficial effects on atherosclerosis, improvement in cardiac function and an obvious reduction in the burden of obesity 66 , but on the other hand, it can be associated with bone loss and fragility fractures 68 . Furthermore, obesity intervention trials have demonstrated that the majority of patients are unable to maintain daily caloric restriction over a long period of time 69 . Intermittent fasting, including alternate-day fasting, full-day fasting patterns and time-restricted eating have been shown to represent viable alternatives to decrease in body weight, also improving lipid and blood pressure control 70 , and reducing markers of oxidative stress and inflammation 71 . Regular physical exercise is the most effective intervention for sarcopenia 72 , defined as the age-related decline in skeletal muscle mass, strength and function, affecting over 50% of individuals at the age of 80 and more. As such, it is a vital component of lifestyle habits associated with healthy aging 73 . Good sleep, regular health monitoring, stress reduction, intellectual and cognitive challenges and non-smoking are other elements necessary to fulfil the goals of healthy aging 74 .

## Asymmetric dimethylarginine

Asymmetric dimethylarginine (ADMA) is an endogenous homologue of L -arginine that inhibits nitric oxide (NO) production by all three known isoforms of NO synthases 75 76 . ADMA is a well-established, independent risk factor for cardiovascular and overall mortality in the general population and in patients with diseases of the cardiovascular, pulmonary, renal, endocrine, and gastrointestinal systems 77 78 79 . Lowering ADMA concentrations in animal models resulted in protection against conditions associated with advanced age, including atherosclerosis, adverse myocardial and vascular remodeling, myocardial and renal ischemia/reperfusion damage, and insulin resistance 80 81 82 83 84 . Observational studies have demonstrated that plasma ADMA concentrations increase with age 85 86 , whereas the nitric oxide:superoxide ratio decreases leading to oxidative stress, inflammation, degenerative changes, insulin resistance, and endothelial dysfunction 87 88 . Animal experiments and pre-clinical studies strongly suggest that ADMA is implicated in biological processes relevant to aging, such as telomerase activity, endothelial senescence, and mitochondrial dysfunction 89 90 91 92 93 94 95 .

In addition to inhibition of NO production, ADMA also “uncouples” NO synthases, which results in the production of superoxide radicals (O _2_^−^ ) instead of NO 96 . The presence of both functional and uncoupled NOS in the cell results in the concomitant production of NO and O _2_^−^ in close vicinity. O _2_^−^ reacts with NO, thus scavenging it and forming the harmful peroxynitrite radical (ONOO ^−^ ). This leads to a vicious cycle that potentiates oxidative stress and drives pathological changes 97 . NO is an endogenous metabolic mitochondrial master modulator that mediates antioxidant protection and regeneration 98 . NO has also been shown to determine mitochondrial biogenesis and bioactivity 99 , as well as maintain neurovascular-neuro energetic coupling and synaptic plasticity and, thus, brain development and cognition. The superoxide anion radicals produced during aging are antagonistic mediators that can induce neurodegeneration and cell death by increasing oxidative stress and damage directly and through NO depletion 100 . Many age-associated pathophysiological processes are modulated by NO, especially arterial stiffness, vascular tone, platelet function, myocardial hypertrophy, and contractility 87 101 .

Independently of the NO/NOS pathway, ADMA was recently reported to promote degeneration and senescence of chondrocytes and cartilage, accelerating progression of osteoarthritis 102 . Furthermore, increased ADMA concentrations have been shown to be associated with disuse-related osteoporosis 103 and with frailty in patients without cardiovascular diseases 104 .

## Targeting aging: place for ADMA and therapeutic apheresis?

**Fig. 2:**
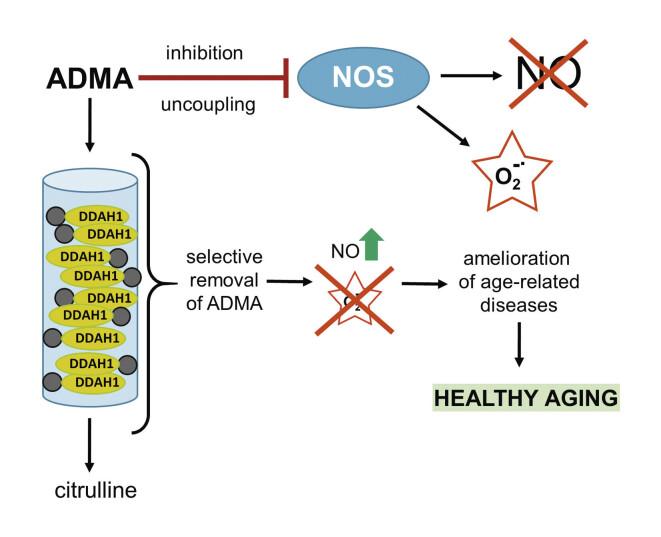
Selective removal of ADMA as a way to healthy aging. ADMA causes both inhibition and uncoupling of NOS, which lead to decreased production of NO and increased release of superoxide radicals. Selective removal of ADMA by its catabolism to citrulline by DDAH1 immobilized on an apheresis column leads to restoration of NO production and redox balance. These ameliorate diseases that are associated with advanced age and promote healthy aging. ADMA: Asymmetric dimethylarginine; DDAH1: Dimethylarginine dimethylaminohydrolase 1; NOS: Nitric oxide synthase; NO: Nitric oxide.

The direct involvement of ADMA at the intersection of molecular pathways involved in the aging process and the association of elevated ADMA concentrations with advanced age-related diseases, together with the strong animal data on beneficial effects of ADMA lowering raise the intriguing possibility of targeting ADMA as an anti-aging therapy. Currently there are no approved clinical approaches to specifically lower ADMA in humans. Driven by this unmet clinical need, we propose the use of an apheresis column with immobilized ADMA-metabolizing enzyme, dimethylarginine dimethylaminohydrolase 1 (DDAH1), to selectively remove ADMA from plasma during apheresis sessions. This strategy should lead to increased production of NO and decreased levels of superoxide radicals, leading to promoting healthy aging through beneficial effects in diseases associated with old age ( Fig. 2 ).

## Conclusions

Selective extracorporeal removal of ADMA during apheresis in the elderly population could be a potential strategy to prevent or delay age-associated disease states, consequently favoring healthy aging. Such approach, using an enzyme immobilized on a column, would be the first example of a novel apheresis principle – enzymatic apheresis – and could pave the way for similar strategies to remove other circulating detrimental substances that cannot be therapeutically targeted otherwise.

## Notice

This article was changed according to the following Erratum on June 5th 2025.

## Erratum

In the above-mentioned article the name of the last author was incomplete. The correct name is Roman N. Rodionov. This was corrected in the online version on 06.06.2025.

## References

[1] Behr L C Simm A Kluttig A 60 years of healthy aging: on definitions, biomarkers, scores and challenges Ageing Res Rev 2023 88 101934 37059401 10.1016/j.arr.2023.101934

[2] Cosco T D Howse K Brayne C Healthy ageing, resilience and wellbeing Epidemiol Psychiatr Sci 2017 26 579 583 28679453 10.1017/S2045796017000324 PMC6998987

[3] Olshansky S J From lifespan to healthspan JAMA 2018 320 1323 1324 30242384 10.1001/jama.2018.12621

[4] Malden D E Mangoni A A Woodman R J Circulating asymmetric dimethylarginine and cognitive decline: a 4-year follow-up study of the 1936 Aberdeen Birth Cohort Int J Geriatr Psychiatry 2020 35 1181 1188 32452069 10.1002/gps.5355

[5] Boger R H Cooke J P Vallance P ADMA: an emerging cardiovascular risk factor Vasc Med 2005 10 S1 S2 10.1177/1358836X0501000101 16444862

[6] Abbasi F Asagmi T Cooke J P Plasma concentrations of asymmetric dimethylarginine are increased in patients with type 2 diabetes mellitus Am J Cardiol 2001 88 1201 1203 11703973 10.1016/s0002-9149(01)02063-x

[7] Boger R H Bode-Boger S M Thiele W Biochemical evidence for impaired nitric oxide synthesis in patients with peripheral arterial occlusive disease Circulation 1997 95 2068 2074 9133517 10.1161/01.cir.95.8.2068

[8] North B J Sinclair D A The intersection between aging and cardiovascular disease Circ Res 2012 110 1097 1108 22499900 10.1161/CIRCRESAHA.111.246876 PMC3366686

[9] Querfurth H W LaFerla F M Alzheimer's disease N Engl J Med 2010 362 329 344 20107219 10.1056/NEJMra0909142

[10] Raisz L G Local and systemic factors in the pathogenesis of osteoporosis N Engl J Med 1988 318 818 828 3281002 10.1056/NEJM198803313181305

[11] Loeser R F Aging and osteoarthritis Curr Opin Rheumatol 2011 23 492 496 21709557 10.1097/BOR.0b013e3283494005 PMC3377970

[12] Gunasekaran U Gannon M Type 2 diabetes and the aging pancreatic beta cell Aging (Albany NY) 2011 3 565 575 21765202 10.18632/aging.100350 PMC3164365

[13] Nalysnyk L Cid-Ruzafa J Rotella P Incidence and prevalence of idiopathic pulmonary fibrosis: review of the literature Eur Respir Rev 2012 21 355 361 23204124 10.1183/09059180.00002512 PMC9487229

[14] Kwon Y H Fingert J H Kuehn M H Primary open-angle glaucoma N Engl J Med 2009 360 1113 1124 19279343 10.1056/NEJMra0804630 PMC3700399

[15] Cortes-Canteli M Iadecola C Alzheimer’s disease and vascular aging: JACC Focus Seminar J Am Coll Cardiol 2020 75 942 951 32130930 10.1016/j.jacc.2019.10.062 PMC8046164

[16] Yuan Q Wang H Gao P Prevalence and risk factors of metabolic-associated fatty liver disease among 73,566 individuals in Beijing, China Int J Environ Res Public Health 2022 19 2096 35206282 10.3390/ijerph19042096 PMC8871878

[17] Bettonte S Berton M Marzolini C Magnitude of drug-drug interactions in special populations Pharmaceutics 2022 14 789 35456623 10.3390/pharmaceutics14040789 PMC9027396

[18] Campisi J Kapahi P Lithgow G J From discoveries in ageing research to therapeutics for healthy ageing Nature 2019 571 183 192 31292558 10.1038/s41586-019-1365-2 PMC7205183

[19] Lopez-Otin C Blasco M A Partridge L The hallmarks of aging Cell 2013 153 1194 1217 23746838 10.1016/j.cell.2013.05.039 PMC3836174

[20] Aman Y Schmauck-Medina T Hansen M Autophagy in healthy aging and disease Nat Aging 2021 1 634 650 34901876 10.1038/s43587-021-00098-4 PMC8659158

[21] Minami S Nakamura S Yoshimori T Rubicon in metabolic diseases and ageing Front Cell Dev Biol 2021 9 816829 35083223 10.3389/fcell.2021.816829 PMC8784836

[22] Northrop J H The influence of the intensity of light on the rate of growth and duration of life of Drosophila J Gen Physiol 1925 9 81 86 19872234 10.1085/jgp.9.1.81 PMC2140775

[23] Zhang Z D Milman S Lin J R Genetics of extreme human longevity to guide drug discovery for healthy ageing Nat Metab 2020 2 663 672 32719537 10.1038/s42255-020-0247-0 PMC7912776

[24] McCay C M Maynard L A Sperling G The Journal of Nutrition. Volume 18 July-December, 1939. Pages 1–13. Retarded growth, life span, ultimate body size and age changes in the albino rat after feeding diets restricted in calories Nutr Rev 1975 33 241 243 1095975 10.1111/j.1753-4887.1975.tb05227.x

[25] Burkle A Moreno-Villanueva M Bernhard J MARK-AGE biomarkers of ageing Mech Ageing Dev 2015 151 2 12 25818235 10.1016/j.mad.2015.03.006

[26] Guo J Huang X Dou L Aging and aging-related diseases: from molecular mechanisms to interventions and treatments Signal Transduct Target Ther 2022 7 391 36522308 10.1038/s41392-022-01251-0 PMC9755275

[27] Sun N Youle R J Finkel T The mitochondrial basis of aging Mol Cell 2016 61 654 666 26942670 10.1016/j.molcel.2016.01.028 PMC4779179

[28] Hayflick L Moorhead P S The serial cultivation of human diploid cell strains Exp Cell Res 1961 25 585 621 13905658 10.1016/0014-4827(61)90192-6

[29] Hayflick L The limited in vitro lifetime of human diploid cell strains Exp Cell Res 1965 37 614 636 14315085 10.1016/0014-4827(65)90211-9

[30] Baker D J Jeganathan K B Cameron J D BubR1 insufficiency causes early onset of aging-associated phenotypes and infertility in mice Nat Genet 2004 36 744 749 15208629 10.1038/ng1382

[31] Baker D J Jin F van Deursen J M The yin and yang of the Cdkn2a locus in senescence and aging Cell Cycle 2008 7 2795 2802 18769141 10.4161/cc.7.18.6687 PMC2987737

[32] Chinta S J Woods G Demaria M Cellular senescence is induced by the environmental neurotoxin paraquat and contributes to neuropathology linked to Parkinson's disease Cell Rep 2018 22 930 940 29386135 10.1016/j.celrep.2017.12.092 PMC5806534

[33] Bussian T J Aziz A Meyer C F Clearance of senescent glial cells prevents tau-dependent pathology and cognitive decline Nature 2018 562 578 582 30232451 10.1038/s41586-018-0543-y PMC6206507

[34] Childs B G Baker D J Wijshake T Senescent intimal foam cells are deleterious at all stages of atherosclerosis Science 2016 354 472 477 27789842 10.1126/science.aaf6659 PMC5112585

[35] Jeon O H Kim C Laberge R M Local clearance of senescent cells attenuates the development of post-traumatic osteoarthritis and creates a pro-regenerative environment Nat Med 2017 23 775 781 28436958 10.1038/nm.4324 PMC5785239

[36] Yosef R Pilpel N Tokarsky-Amiel R Directed elimination of senescent cells by inhibition of BCL-W and BCL-XL Nat Commun 2016 7 11190 27048913 10.1038/ncomms11190 PMC4823827

[37] Childs B G Baker D J Kirkland J L Senescence and apoptosis: dueling or complementary cell fates? EMBO Rep 2014 15 1139 1153 25312810 10.15252/embr.201439245 PMC4253488

[38] Ovadya Y Landsberger T Leins H Impaired immune surveillance accelerates accumulation of senescent cells and aging Nat Commun 2018 9 5435 30575733 10.1038/s41467-018-07825-3 PMC6303397

[39] Fumagalli M Rossiello F Mondello C Stable cellular senescence is associated with persistent DDR activation PLoS One 2014 9 e110969 25340529 10.1371/journal.pone.0110969 PMC4207795

[40] d'Adda di Fagagna F Reaper F M Clay-Farrace L A DNA damage checkpoint response in telomere-initiated senescence Nature 2003 426 194 198 14608368 10.1038/nature02118

[41] Mallette F A Ferbeyre G The DNA damage signaling pathway connects oncogenic stress to cellular senescence Cell Cycle 2007 6 1831 1836 17671427 10.4161/cc.6.15.4516

[42] Beausejour C M Krtolica A Galimi F Reversal of human cellular senescence: roles of the p53 and p16 pathways EMBO J 2003 22 4212 4222 12912919 10.1093/emboj/cdg417 PMC175806

[43] Dulic V Beney G E Frebourg G Uncoupling between phenotypic senescence and cell cycle arrest in aging p21-deficient fibroblasts Mol Cell Biol 2000 20 6741 6754 10958672 10.1128/mcb.20.18.6741-6754.2000 PMC86196

[44] Herbig U Jobling W A Chen B P Telomere shortening triggers senescence of human cells through a pathway involving ATM, p53, and p21(CIP1), but not p16(INK4a) Mol Cell 2004 14 501 513 15149599 10.1016/s1097-2765(04)00256-4

[45] D'Arcy M S Cell death: a review of the major forms of apoptosis, necrosis and autophagy Cell Biol Int 2019 43 582 592 30958602 10.1002/cbin.11137

[46] Islam M A Sooro M A Zhang P Autophagic regulation of p62 is critical for cancer therapy Int J Mol Sci 2018 19 1405 29738493 10.3390/ijms19051405 PMC5983640

[47] Barbosa M C Grosso R A Fader C M Hallmarks of aging: an autophagic perspective Front Endocrinol (Lausanne) 2018 9 790 30687233 10.3389/fendo.2018.00790 PMC6333684

[48] Simonsen A Cumming R C Brech A Promoting basal levels of autophagy in the nervous system enhances longevity and oxidant resistance in adult Drosophila Autophagy 2008 4 176 184 18059160 10.4161/auto.5269

[49] Ulgherait M Rana A Rera M AMPK modulates tissue and organismal aging in a non-cell-autonomous manner Cell Rep 2014 8 1767 1780 25199830 10.1016/j.celrep.2014.08.006 PMC4177313

[50] Juhasz G Erdi B Sass M Atg7-dependent autophagy promotes neuronal health, stress tolerance, and longevity but is dispensable for metamorphosis in Drosophila Genes Dev 2007 21 3061 3066 18056421 10.1101/gad.1600707 PMC2081972

[51] Hars E S Qi H Ryazanov A G Autophagy regulates ageing in C. elegans Autophagy 2007 3 93 95 17204841 10.4161/auto.3636

[52] Pyo J O Yoo S M Ahn H H Overexpression of Atg5 in mice activates autophagy and extends lifespan Nat Commun 2013 4 2300 23939249 10.1038/ncomms3300 PMC3753544

[53] Fernandez A F Sebti S Wei Y Disruption of the beclin 1-BCL2 autophagy regulatory complex promotes longevity in mice Nature 2018 558 136 140 29849149 10.1038/s41586-018-0162-7 PMC5992097

[54] Cassidy L D Young AR J Young CN J Temporal inhibition of autophagy reveals segmental reversal of ageing with increased cancer risk Nat Commun 2020 11 307 31949142 10.1038/s41467-019-14187-x PMC6965206

[55] Tabibzadeh S Role of autophagy in aging: the good, the bad, and the ugly Aging Cell 2023 22 e13753 36539927 10.1111/acel.13753 PMC9835585

[56] Ortega M A Fraile-Martinez O de Leon-Oliva D Autophagy in its (proper) context: molecular basis, biological relevance, pharmacological modulation, and lifestyle medicine Int J Biol Sci 2024 20 2532 2554 38725847 10.7150/ijbs.95122 PMC11077378

[57] Wiley C D Velarde M C Lecot P Mitochondrial dysfunction induces senescence with a distinct secretory phenotype Cell Metab 2016 23 303 314 26686024 10.1016/j.cmet.2015.11.011 PMC4749409

[58] Bratic A Larsson N G The role of mitochondria in aging J Clin Invest 2013 123 951 957 23454757 10.1172/JCI64125 PMC3582127

[59] Indo H P Yen H C Nakanishi I A mitochondrial superoxide theory for oxidative stress diseases and aging J Clin Biochem Nutr 2015 56 1 7 25834301 10.3164/jcbn.14-42 PMC4306659

[60] Genova M L Lenaz G The interplay between respiratory supercomplexes and ROS in aging Antioxid Redox Signal 2015 23 208 238 25711676 10.1089/ars.2014.6214

[61] Zhang Y Ikeno Y Qi W Mice deficient in both Mn superoxide dismutase and glutathione peroxidase-1 have increased oxidative damage and a greater incidence of pathology but no reduction in longevity J Gerontol A Biol Sci Med Sci 2009 64 1212 1220 19776219 10.1093/gerona/glp132 PMC2781787

[62] Kwon M J Lee K Y Lee H W SOD3 Variant, R213G, altered SOD3 function, leading to ROS-mediated inflammation and damage in multiple organs of premature aging mice Antioxid Redox Signal 2015 23 985 999 25927599 10.1089/ars.2014.6035

[63] Mossad O Batut B Yilmaz B Gut microbiota drives age-related oxidative stress and mitochondrial damage in microglia via the metabolite N(6)-carboxymethyllysine Nat Neurosci 2022 25 295 305 35241804 10.1038/s41593-022-01027-3

[64] Yeung SS Y Kwan M Woo J Healthy diet for healthy aging Nutrients 2021 13 4310 34959862 10.3390/nu13124310 PMC8707325

[65] Kiefte-de Jong J C Mathers J C Franco O H Nutrition and healthy ageing: the key ingredients Proc Nutr Soc 2014 73 249 259 24503212 10.1017/S0029665113003881

[66] Le Couteur D G Simpson S J 90th Anniversary commentary: caloric restriction effects on aging J Nutr 2018 148 1656 1659 30281103 10.1093/jn/nxy146

[67] Dominguez L J Veronese N Baiamonte E Healthy aging and dietary patterns Nutrients 2022 14 889 35215539 10.3390/nu14040889 PMC8879056

[68] Shanley D P Kirkwood T B Caloric restriction does not enhance longevity in all species and is unlikely to do so in humans Biogerontology 2006 7 165 168 16858629 10.1007/s10522-006-9006-1

[69] Nowosad K Sujka M Effect of various types of intermittent fasting (IF) on weight loss and improvement of diabetic parameters in human Curr Nutr Rep 2021 10 146 154 33826120 10.1007/s13668-021-00353-5 PMC8102292

[70] Vasim I Majeed C N DeBoer M D Intermittent fasting and metabolic health Nutrients 2022 14 631 35276989 10.3390/nu14030631 PMC8839325

[71] Johnson J B Summer W Cutler R G Alternate day calorie restriction improves clinical findings and reduces markers of oxidative stress and inflammation in overweight adults with moderate asthma Free Radic Biol Med 2007 42 665 674 17291990 10.1016/j.freeradbiomed.2006.12.005 PMC1859864

[72] Shen Y Shi Q Nong K Exercise for sarcopenia in older people: a systematic review and network meta-analysis J Cachexia Sarcopenia Muscle 2023 14 1199 1211 37057640 10.1002/jcsm.13225 PMC10235889

[73] Landi F Liperoti R Fusco D Prevalence and risk factors of sarcopenia among nursing home older residents J Gerontol A Biol Sci Med Sci 2012 67 48 55 21393423 10.1093/gerona/glr035

[74] Furrer R Handschin C Lifestyle vs. pharmacological interventions for healthy aging Aging (Albany NY) 2020 12 5 7 31937689 10.18632/aging.102741 PMC6977688

[75] Tran C T Leiper J M Vallance P The DDAH/ADMA/NOS pathway Atheroscler Suppl 2003 4 33 40 14664901 10.1016/s1567-5688(03)00032-1

[76] Vallance P Leone A Calver A Endogenous dimethylarginine as an inhibitor of nitric oxide synthesis J Cardiovasc Pharmacol 1992 20 S60 S62 1282988 10.1097/00005344-199204002-00018

[77] Meinitzer A Seelhorst U Wellnitz B Asymmetrical dimethylarginine independently predicts total and cardiovascular mortality in individuals with angiographic coronary artery disease (the Ludwigshafen Risk and Cardiovascular Health study) Clin Chem 2007 53 273 283 17185364 10.1373/clinchem.2006.076711

[78] Schlesinger S Sonntag S R Lieb W Asymmetric and symmetric dimethylarginine as risk markers for total mortality and cardiovascular outcomes: a systematic review and meta-analysis of prospective studies PLoS One 2016 11 e0165811 27812151 10.1371/journal.pone.0165811 PMC5094762

[79] Zoccali C Bode-Boger S Mallamaci F Plasma concentration of asymmetrical dimethylarginine and mortality in patients with end-stage renal disease: a prospective study Lancet 2001 358 2113 2117 11784625 10.1016/s0140-6736(01)07217-8

[80] Jacobi J Maas R Cardounel A J Dimethylarginine dimethylaminohydrolase overexpression ameliorates atherosclerosis in apolipoprotein E-deficient mice by lowering asymmetric dimethylarginine Am J Pathol 2010 176 2559 2570 20348244 10.2353/ajpath.2010.090614 PMC2861120

[81] Sydow K Mondon C E Schrader J Dimethylarginine dimethylaminohydrolase overexpression enhances insulin sensitivity Arterioscler Thromb Vasc Biol 2008 28 692 697 18239148 10.1161/ATVBAHA.108.162073 PMC3165027

[82] Nakayama Y Ueda S Yamagishi S Asymmetric dimethylarginine accumulates in the kidney during ischemia/reperfusion injury Kidney Int 2014 85 570 578 24107853 10.1038/ki.2013.398 PMC3944656

[83] Stuhlinger M C Conci E Haubner B J Asymmetric dimethyl l-arginine (ADMA) is a critical regulator of myocardial reperfusion injury Cardiovasc Res 2007 75 417 425 17559823 10.1016/j.cardiores.2007.04.030

[84] Konishi H Sydow K Cooke J P Dimethylarginine dimethylaminohydrolase promotes endothelial repair after vascular injury J Am Coll Cardiol 2007 49 1099 1105 17349891 10.1016/j.jacc.2006.10.068

[85] Boger R H Sullivan L M Schwedhelm E Plasma asymmetric dimethylarginine and incidence of cardiovascular disease and death in the community Circulation 2009 119 1592 1600 19289633 10.1161/CIRCULATIONAHA.108.838268 PMC2742491

[86] Schulze F Maas R Freese R Determination of a reference value for N(G), N(G)-dimethyl-l-arginine in 500 subjects Eur J Clin Invest 2005 35 622 626 16178881 10.1111/j.1365-2362.2005.01561.x

[87] Sverdlov A L Ngo D T Chan W P Aging of the nitric oxide system: are we as old as our NO? J Am Heart Assoc 2014 3 e000973 25134680 10.1161/JAHA.114.000973 PMC4310385

[88] El Assar M Angulo J Santos-Ruiz M Asymmetric dimethylarginine (ADMA) elevation and arginase up-regulation contribute to endothelial dysfunction related to insulin resistance in rats and morbidly obese humans J Physiol 2016 594 3045 3060 26840628 10.1113/JP271836 PMC4887698

[89] Perticone F Sciacqua A Maio R Endothelial dysfunction, ADMA and insulin resistance in essential hypertension Int J Cardiol 2010 142 236 241 19168237 10.1016/j.ijcard.2008.12.131

[90] Juonala M Viikari J S Alfthan G Brachial artery flow-mediated dilation and asymmetrical dimethylarginine in the cardiovascular risk in young Finns study Circulation 2007 116 1367 1373 17724260 10.1161/CIRCULATIONAHA.107.690016

[91] Bode-Boger S M Scalera F Martens-Lobenhoffer J Asymmetric dimethylarginine (ADMA) accelerates cell senescence Vasc Med 2005 10 S65 S71 16444871 10.1177/1358836X0501000110

[92] Xiong Y He Y L Li X M Endogenous asymmetric dimethylarginine accumulation precipitates the cardiac and mitochondrial dysfunctions in type 1 diabetic rats Eur J Pharmacol 2021 902 174081 33901463 10.1016/j.ejphar.2021.174081

[93] Xiong Y Hai C X Fang W J Endogenous asymmetric dimethylarginine accumulation contributes to the suppression of myocardial mitochondrial biogenesis in type 2 diabetic rats Nutr Metab (Lond) 2020 17 72 32855652 10.1186/s12986-020-00486-4 PMC7445927

[94] Scalera F Borlak J Beckmann B Endogenous nitric oxide synthesis inhibitor asymmetric dimethyl l-arginine accelerates endothelial cell senescence Arterioscler Thromb Vasc Biol 2004 24 1816 1822 15308550 10.1161/01.ATV.0000141843.77133.fc

[95] Adelibieke Y Shimizu H Muteliefu G Indoxyl sulfate induces endothelial cell senescence by increasing reactive oxygen species production and p53 activity J Ren Nutr 2012 22 86 89 22200421 10.1053/j.jrn.2011.10.027

[96] Antoniades C Shirodaria C Leeson P Association of plasma asymmetrical dimethylarginine (ADMA) with elevated vascular superoxide production and endothelial nitric oxide synthase uncoupling: implications for endothelial function in human atherosclerosis Eur Heart J 2009 30 1142 1150 19297385 10.1093/eurheartj/ehp061

[97] Forstermann U Munzel T Endothelial nitric oxide synthase in vascular disease: from marvel to menace Circulation 2006 113 1708 1714 16585403 10.1161/CIRCULATIONAHA.105.602532

[98] Bode-Boger S M Muke J Surdacki A Oral l-arginine improves endothelial function in healthy individuals older than 70 years Vasc Med 2003 8 77 81 14518608 10.1191/1358863x03vm474oa

[99] Nisoli E Clementi E Paolucci C Mitochondrial biogenesis in mammals: the role of endogenous nitric oxide Science 2003 299 896 899 12574632 10.1126/science.1079368

[100] Lourenco C F Ledo A Barbosa R M Neurovascular-neuroenergetic coupling axis in the brain: master regulation by nitric oxide and consequences in aging and neurodegeneration Free Radic Biol Med 2017 108 668 682 28435052 10.1016/j.freeradbiomed.2017.04.026

[101] Chirkov Y Y Horowitz J D Impaired tissue responsiveness to organic nitrates and nitric oxide: a new therapeutic frontier? Pharmacol Ther 2007 116 287 305 17765975 10.1016/j.pharmthera.2007.06.012

[102] Wu Y Shen S Chen J Metabolite asymmetric dimethylarginine (ADMA) functions as a destabilization enhancer of SOX9 mediated by DDAH1 in osteoarthritis Sci Adv 2023 9 eade5584 36753544 10.1126/sciadv.ade5584 PMC9908022

[103] Xie Z Hou L Shen S Mechanical force promotes dimethylarginine dimethylaminohydrolase 1-mediated hydrolysis of the metabolite asymmetric dimethylarginine to enhance bone formation Nat Commun 2022 13 50 35013196 10.1038/s41467-021-27629-2 PMC8748781

[104] Alonso-Bouzon C Carcaillon L Garcia-Garcia F J Association between endothelial dysfunction and frailty: the Toledo study for healthy aging Age (Dordr) 2014 36 495 505 23959520 10.1007/s11357-013-9576-1 PMC3889911

