# Correction: Asymmetric Dimethylarginine: A Never-Aging Story

**DOI:** 10.1055/a-2626-3601

**Published:** 2025-06-10

**Authors:** Natalia Jarzebska, Stefan R. Bornstein, Sergey Tselmin, Ulrich Julius, Barbara Cellini, Richard Siow, Mike Martin, Rajeshwar P. Mookerjee, Arduino A. Mangoni, Norbert Weiss, Roman N. Rodionov

**Affiliations:** 1 Department of Internal Medicine III, University Hospital Carl Gustav Carus at the Technische Universität Dresden, Dresden, Germany; 2 School of Cardiovascular and Metabolic Medicine and Sciences, Faculty of Life Sciences & Medicine, King’s College London, London, United Kingdom of Great Britain and Northern Ireland; 3 Department of Medicine and Surgery, University of Perugia, Perugia, Italy; 4 Ageing Research at King’s (ARK), King’s College London, London, United Kingdom of Great Britain and Northern Ireland; 5 Department of Physiology, Anatomy and Genetics, Medical Sciences Division, University of Oxford, Oxford, United Kingdom of Great Britain and Northern Ireland; 6 Department of Psychology, University of Zurich, Zurich, Switzerland; 7 Healthy Longevity Center, University of Zurich, Zurich, Switzerland; 8 Institute of Liver and Digestive Health, University College London, London, United Kingdom of Great Britain and Northern Ireland; 9 Department of Clinical Pharmacology, College of Medicine and Public Health, Flinders University and Flinders Medical Centre, Adelaide, Australia



In the above-mentioned article the name of the last author was incomplete. The correct name is Roman N. Rodionov. This was corrected in the online version on 06.06.2025.

